# No association between HMGB1 polymorphisms and cancer risk: evidence from a meta-analysis

**DOI:** 10.1042/BSR20180658

**Published:** 2018-09-05

**Authors:** Xing-yan Li, Chun-hua Liang, Ye-jing Yang, Lei Liu, Yong-jun Du, Hong-suo Liang, Lin Li, Bo Zhang, Jian-min Li, Jin-min Zhao

**Affiliations:** 1 Department of Orthopedics, The Third Affiliated Hospital of Guangxi Medical University, Nanning, Guangxi 530031, China; 2 Department of Orthopedics, The First Affiliated Hospital of Guangxi Medical University, Nanning, Guangxi 530021, China

**Keywords:** cancer, High mobility group box 1, meta analysis, single nucleotide polymorphisms

## Abstract

The aim of the present study was to determine whether High mobility group box 1 (HMGB1) polymorphism was associated with cancer susceptibility. PubMed, Embase, and ISI Web of Science were extensively searched without language restriction. Data were extracted using a standardized data collection sheet after two reviewers scanned studies independently. The association between HMGB1 polymorphism and cancer risks was indicated as odds ratio (OR) along with its related 95% confidence interval (95%CI). Meta-analysis was conducted via RevMan 5.3 software. A total of ten studies comprising 4530 cases and 5167 controls were included in our study. Meta-analysis revealed no statistical association between *rs1045411, rs1360485, rs1412125*, or *rs2249825* polymorphisms in *HMGB1* gene and risk of cancer, either did subgroup analysis of *rs1045411* stratified by cancer types and ethnic groups. Our results revealed no statistical association between current four polymorphism loci and cancer risks, suggesting that the attempt of applying HMGB1 variants as a therapeutic target or a prognosis predictor might still require a second thought. However, HMGB1 is deemed to play pleiotropic roles in cancers, we strongly call for large-scale studies with high evidence level to uncover the exact relationship between *HMGB1* gene variants and cancer progression.

## Introduction

High mobility group box 1 (HMGB1), also known as HMG1 [1], is a highly conserved, ubiquitously expressed single polypeptide in all mammalian eukaryotic cells [2,3]. It is located in the nucleus, acting as a DNA chaperone, chromosome guardian, autophagy sustainer, and protector from apoptotic cell death [4,5]. Meanwhile it also has the ability to shuttle to cytoplasma and activate autophage through interacting with Beclin-1 [6], as well as to translocate to the extracellular medium to act as a prototypic damage-associated molecular pattern (DAMP) molecule when passively released from dead, dying or injured cells, or when actively secreted from immune cells or cancer cells in response to exogenous and endogenous stimuli, thus is deemed to play a significant role in an impressive number of medical conditions [7,8].

Till now, studies have correlated the HMGB1 protein to cancer progression, especially invasion and metastasis [9]. In the experimental setting, inhibition of HMGB1 release diminish ATP production and retard tumor growth [10]. Overexpression of HMGB1 in tumor tissue and increased HMGB1 serum level are near-universal in virtually every examined type of cancer, including colon carcinoma [11–13], hepatoma [14,15], breast cancer (BC) [16], pancreatic cancer [11], melanoma [17], ovarian cancer [18], and mesothelioma [19,20], indicating a carcinogenic role of HMGB1.

Intriguingly, HMGB1 may also function as a tumor suppressor. It has been found to increase the binding affinity of many sequence-specific transcription factors to their cognate DNA, such as p53, p73, the retinoblastoma protein (Rb), nuclear factor-κB (NF-κB), and the estrogen receptor [21]. The overexpression of HMGB1 inhibits Rb-positive BC cells growth *in vitro*, also preventing growth in *in vivo* tumor models [22,23]. Current knowledge is that HMGB1 may play paradoxical roles in cancer, although its exact mechanism and physiological meaning still remain to be further investigated.

Cancer development is a multistep progress. Chromosomal instability, which could be driven by complex genetic and physiological variations as well as environmental factors, is considered to be central to the pathogenesis of malignancies [24,25]. Loss of HMGB1 could reduce telomerase activity, decrease telomere length, and increase chromosomal instability [4,5,26]. Thus, understanding the molecular bases might be important for exploring the exact role of HMGB1 in cancer, as well as developing new diagnostic biomarkers and identifying new therapeutic targets.

Although flourishing numbers of researches have demonstrated that the existence of HMGB1 polymorphism made it possible for affecting the susceptibility [27–36], prognosis [15,37,38] as well as treatment response of malignancies [39], the correlation between HMGB1 polymorphism and risks of cancer remains controversial. Hence, we conducted a comprehensive study based on current research to better understand the association between *HMGB1* gene polymorphisms and cancer risks.

## Materials and methods

This systematic review was conducted in accordance with the Preferred Reporting Items for Systematic Reviews and Meta-Analyses (PRISMA) guidelines [40].

### Literature search strategy

We systemically searched PubMed, Embase, and ISI Web of Science (from their commencements to May 2018) with no language restrictions, for studies in humans of correlations between HMGB1 polymorphisms and cancer risks. The following terms were used for English online databases: (Single Nucleotide Polymorphism or polymorphism or SNP or SNPs or ‘Polymorphism, Single Nucleotide’[Mesh]) and (HMGB1 or high mobility group box 1 or HMG-1). To broaden our search, references of related reviews were also manually scrutinized.

### Inclusion and exclusion criteria

In this meta-analysis, publications were eligible if they fulfilled the following criteria: (i) case–control study or cohort study design; (ii) evaluated the association of the genetic polymorphisms of HMGB1 with the risk of cancer; (iii) displayed outcomes in the form of odds ratio (OR) with 95% confidence interval (95%CI) or provided sufficient genotypic and/or allelic information for estimating OR with 95%CI.

The exclusion criteria included: (i) duplicated studies using the same population or overlapping database; (ii) non-human research; (iii) studies that were case reports, editorials, reviews, letters, and comments.

### Quality assessment of included studies

The Newcastle–Ottawa Scale (NOS) [41] for assessing the quality of non-randomized studies was employed to judge the included studies on three broad perspectives: the selection of the study groups; the comparability of the groups; and the ascertainment of either the exposure or outcome of interest for case–control or cohort studies, respectively. On a scale from 0 to 9, studies scoring 0–3 points, 4–6 points, or 7–9 points were considered to have a high, moderate, or low risk of bias, respectively. Two reviewers (X.-y.L. and C.-h.L.) assessed the risk of bias amongst studies independently and compared the results of quality assessment afterward. In case of discrepancy regarding the quality assessment, consensus was reached through discussion with a third reviewer (J.-m.Z.).

### Data extraction

Study selection was achieved by two investigators independently based on the pre-decided inclusion criteria, any dispute was solved by discussion. Data from the selected studies were collected using a standard data collection sheet including first author and year of publication, SNP loci, country and ethnicity of participants, sample size, source of cases, number of cases and controls for each genotype, and Hardy–Weinberg equilibrium (HWE).

### Statistical analysis

Deviation from HWE was evaluated by using Chi-square test to assess goodness-of-fit in control subjects of each included study. The magnitude of association between HMGB1 polymorphisms and risk of cancer was expressed as OR along with the associated 95%CI. In order to avoid an inflated Type I error rate, we did not perform any prior assumptions about the genetic model of inherence in advance. The most plausible genetic model for HMGB1 polymorphisms in the risk of cancer was identified by a model-free approach recommended by Thakkinstian et al. [42]. If A variant was the gene of interest that could possibly result in an increased or decreased risk of cancer, then OR1, OR2, and OR3 were calculated for genotypes AA compared with aa, Aa compared with aa, AA compared with Aa for each polymorphism to capture the magnitude of genetic effect and to decipher the most plausible genetic model. Then the most reasonable genetic model of inherence was ascertained according to the associations between the three pairwise comparisons as follows:

1. Recessive model: if OR1 = OR3≠1 and OR2 = 1;
2. Dominant model: if OR1 = OR2≠1 and OR3 = 1;
3. Complete over-dominant model: if OR1 = 1, OR2 = 1/OR3≠1;
4. Codominant model: if OR1 > OR2 > 1 and OR1 > OR3 > 1, or OR1 < OR2 < 1 and OR1 < OR3 < 1.

After that the underlying genetic model was confirmed, the counts of each genotype were collapsed into two categories to obtain the merged results. The between-study heterogeneity was assessed using the *Q*-statistical test and *I^2^* test. The random-effect model and fixed-effect model were used for data combination in the presence (*P*<0.1, *I^2^*> 50%) or absence of heterogeneity (*P*>0.1, *I^2^* < 50% indicates acceptable heterogeneity), respectively [43].

In case of statistically significant heterogeneity across studies, subgroup analyses by ethnicity, type of cancer were performed to find the possible source of heterogeneity. The Leave-one-out sensitivity analysis was conducted by removing each study in turn and reassessing the resulting effect on the overall effect. Egger’s regression test and Begg’s rank correlation test [44] were used to estimate the publication bias (Stata version 12.0, Stata Corp LP, U.S.A.). Forest plots and funnel plots were generated using RevMan 5.3 software (Copenhagen: The Nordic Cochrane Centre, The Cochrane Collaboration, 2014).

## Results

### Literature search results

**Figure 1:**
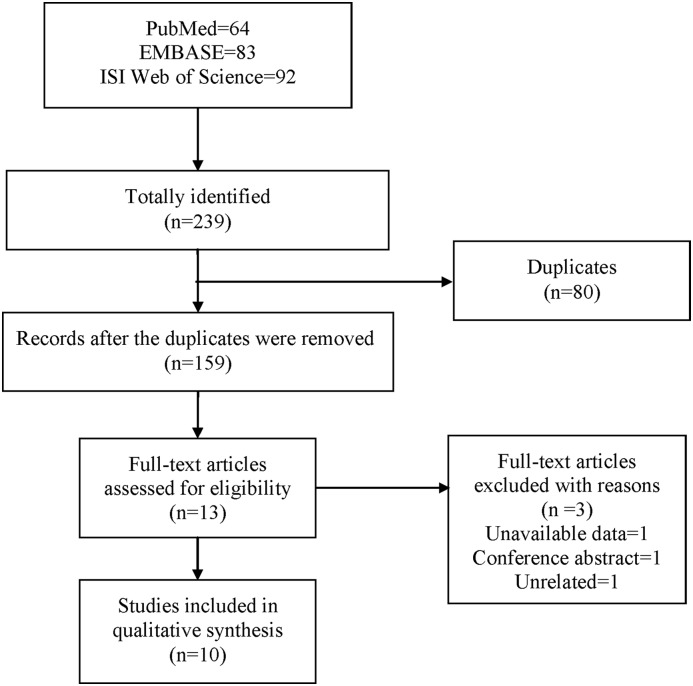
Flowchart of literature search

An initial search yielded 239 potential citations, amongst which 64 from PubMed, 83 from Embase, and 92 from ISI Web of Science, respectively. Eighty citations were deleted because they were duplicates. After screening titles and abstracts, thirteen studies were selected and retrieved for full-text assessment based on predetermined inclusion criteria, amongst which one included unavailable data, one was conference abstract, and one was unrelated. Finally, a total of ten studies [27–36] were considered eligible for inclusion in this meta-analysis. The literature screening proceed was presented in Figure 1.

### Study characteristics

As Table 1 included main characteristics of included ten studies, nine [27–35], six [27,28,30,33,35,36], nine [27,28,30–36], nine [27–35] studies were respectively identified for investigating relationships between *rs1045411, rs1360485, rs1412125, rs2249825* polymorphism and risks of cancers, covering patients of lung cancer (LC) [27,36], oral squamous cell carcinoma (OSCC) [28,29], hepatocellular carcinoma (HCC) [30,31], colorectal cancer (CRC) [32], uterine cervical cancer (UCC) [33], and BC [34,35], amongst which nine studies included Han patients [27,28,30–36] and one focussed on Caucasian cases [29]. The sample sizes of included studies ranged from 193 to 1972, and all the included studies were published between 2015 and 2018. A total number of 4530 cases and 5167 controls were included in this meta-analysis.

**Table 1:** Main characteristics of included studies

Study	Polymorphism	Country	Ethnicity	Sample size (case/control)	Source of cases	Case			Control			HWE
						WT	HT	MT	WT	HT	MT	
Hu W. (2017) [27]	rs1045411 (C>T)	China	Han	372/379	LC	130	54	6	109	71	7	0.542
Huang B.F. (2018) [35]	rs1045411 (C>T)	China	Han	313/217	BC	200	90	23	132	75	10	0.988
Lin C.W. (2017) [28]	rs1045411 (C>T)	Taiwan	Han	1200/772	OSCC	507	226	39	723	411	66	0.753
Supic G. (2015) [29]	rs1045411 (C>T)	Belgrade	Caucasian	93/100	OSCC	48	40	5	64	30	6	0.632
Wang B. (2016) [30]	rs1045411 (C>T)	Taiwan	Han	324/695	HCC	223	89	12	425	239	31	0.939
Wang D. (2017) [31]	rs1045411 (C>T)	China	Han	540/540	HCC	349	158	33	405	127	8	0.859
Wang J.X. (2016) [32]	rs1045411 (C>T)	China	Han	240/480	CRC	144	82	14	268	194	18	0.057
Wu H.H. (2016) [33]	rs1045411 (C>T)	Taiwan	Han	502/305	UCC	117	69	11	204	91	10	0.999
Yue L. (2016) [34]	rs1045411 (C>T)	China	Han	524/518	BC	373	138	13	389	124	5	0.359
Hu W. (2017) [27]	rs1360485 (T>C)	China	Han	372/379	LC	124	56	10	107	68	12	0.964
Huang B.F. (2018) [35]	rs1360485 (T>C)	China	Han	313/217	BC	191	99	23	131	71	15	0.467
Jiang M. (2018) [36]	rs1360485 (T>C)	China	Han	850/733	LC	579	245	26	464	238	31	0.998
Lin C.W. (2017) [28]	rs1360485 (T>C)	Taiwan	Han	1200/772	OSCC	452	273	47	682	440	78	0.826
Wang B. (2016) [30]	rs1360485 (T>C)	Taiwan	Han	324/695	HCC	192	188	14	399	257	39	0.961
Wu H.H. (2016) [33]	rs1360485 (T>C)	Taiwan	Han	502/305	UCC	111	73	13	183	110	12	0.662
Hu W. (2017) [27]	rs1412125 (T>C)	China	Han	372/379	LC	109	70	11	107	69	11	0.999
Huang B.F. (2018) [35]	rs1412125 (T>C)	China	Han	313/217	BC	172	122	21	132	70	15	0.412
Jiang M. (2018) [36]	rs1412125 (T>C)	China	Han	850/733	LC	511	296	43	396	290	47	0.439
Lin C.W. (2017) [28]	rs1412125 (T>C)	Taiwan	Han	1200/772	OSCC	438	274	60	649	457	94	0.560
Wang B. (2016) [30]	rs1412125 (T>C)	Taiwan	Han	324/695	HCC	173	130	21	374	275	46	0.892
Wang D. (2017) [31]	rs1412125 (T>C)	China	Han	540/540	HCC	273	216	51	290	205	45	0.594
Wang J.X. (2016) [32]	rs1412125 (T>C)	China	Han	240/480	CRC	126	103	11	270	195	15	0.015
Wu H.H. (2016) [33]	rs1412125 (T>C)	Taiwan	Han	502/305	UCC	83	97	17	173	114	18	0.991
Yue L. (2016) [34]	rs1412125 (T>C)	China	Han	524/518	BC	281	213	30	300	193	25	0.693
Hu W. (2017) [27]	rs2249825 (G>C)	China	Han	372/379	LC	142	46	2	133	50	4	0.962
Huang B.F. (2018) [35]	rs2249825 (G>C)	China	Han	313/217	BC	214	91	8	163	48	6	0.573
Lin C.W. (2017) [28]	rs2249825 (G>C)	Taiwan	Han	1200/772	OSCC	573	183	16	852	316	32	0.917
Supic G. (2015) [29]	rs2249825 (G>C)	Belgrade	Caucasian	93/100	OSCC	63	27	3	66	26	8	0.096
Wang B. (2016) [30]	rs2249825 (G>C)	Taiwan	Han	324/695	HCC	235	83	6	521	163	11	0.911
Wang D. (2017) [31]	rs2249825 (G>C)	China	Han	540/540	HCC	349	168	23	354	170	16	0.716
Wang J.X. (2016) [32]	rs2249825 (G>C)	China	Han	240/480	CRC	131	94	15	364	98	18	0.005
Wu H.H. (2016) [33]	rs2249825 (G>C)	Taiwan	Han	502/305	UCC	128	63	6	227	73	5	0.952
Yue L. (2016) [34]	rs2249825 (G>C)	China	Han	524/518	BC	462	61	1	432	83	3	0.899

Abbreviation: WT/HT/MT, number of genotype of wild-type, heterozygote, and variant (mutant).

The risk of bias amongst studies was assessed using NOS scale (Table 2). Studies included in this work were considered to be of moderate to high quality, amongst which four [27,28,30,35], three [29,34,36], three [31–33] studies gained 5, 6, 7 stars, respectively.

**Table 2:** Quality assessment of included studies

Item/study	Hu W. (2017) [27]	Huang B.F. (2018) [35]	Jiang M. (2018) [36]	Lin C.W. (2017) [28]	Supic G. (2015) [29]	Wang B. (2016) [30]	Wang D. (2017) [31]	Wang J.X. (2016) [32]	Wu H.H. (2016) [33]	Yue L. (2016) [34]
Adequate definition of cases	*	*	*	*	*	*	*	*	*	*
Representativeness of cases	-	-	-	-	-	-	-	-	*	-
Selection of control subjects	-	-	-	-	-	-	-	-	*	-
Definition of control subjects	*	*	*	*	*	*	*	*	*	*
Control for important factor or additional factor	-	-	*	-	*	-	**	**	-	*
Exposure assessment	*	*	*	*	*	*	*	*	*	*
Same method of ascertainment for all subjects	*	*	*	*	*	*	*	*	*	*
Non-response rate	*	*	*	*	*	*	*	*	*	*

A study could be awarded a maximum of one star (*) for each item except, ‘Control for important factor or additional factor’.

The definition/explanation of each column of the NOS is available from http://www.ohri.ca/programs/clinical_epidemiology/oxford.asp.

### Meta-analyses

**Figure 2:**
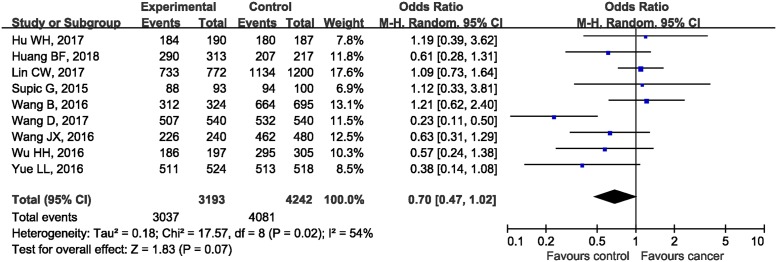
Forest plot of rs1045411 in *HMGB1* gene and risk of cancer using a dominant model

Before combining data from each individual study included, we made testable hypotheses about the most appropriate genetic model of inherence. For the association between *rs1360485* in HMGB1 and cancer risks, OR1 (1.02, 95%CI: 0.80, 1.28; *P*=0.90), OR2 (1.07, 95%CI: 0.94, 1.22; *P*=0.31), and OR3 (1.08, 95%CI: 0.92, 1.62; *P*=0.36) were all statistically insignificant, which suggested no potential association between *rs1360485* and risk of cancer. Null associations were also found for *rs1412125* (OR1: 1.16, 95%CI: 0.98, 1.38; OR2: 1.08, 95%CI: 0.98, 1.19; OR3: 1.10, 95%CI: 0.96, 1.25), *rs2249825* (OR1: 1.11, 95%CI: 0.82, 1.50; OR2: 1.07, 95%CI: 0.96, 1.20; OR3: 0.84, 95%CI: 0.65, 1.08) polymorphisms and cancer risks. However, in terms of *rs1045411*, the estimated OR1 (CC/TT: 0.77, 95%CI: 0.61, 0.97; *P*=0.03) and OR2 (CT/TT: 0.69, 95%CI: 0.54, 0.89; *P*=0.004) were both statistically significant, whereas the estimated OR3 (CC/CT: 1.07, 95%CI: 0.97, 1.18; *P*=0.20) was insignificant, suggesting that the dominant model was the most plausible genetic model for meta-analysis. When using the dominant model, the counts of genotypes of CC and CT groups were combined together and compared with TT groups. Since there was moderate heterogeneity amongst included studies (*P*=0.02, *I^2^* = 54%), the random-effect model was used for statistical analysis. The combined ORs showed that there was no statistically significant association between *rs1045411* in *HMGB1* gene and risk of cancer (OR 0.70, 95%CI: 0.47, 1.02; *P*=0.07) (Figure 2).

### Subgroup analysis and publication bias

**Figure 3:**
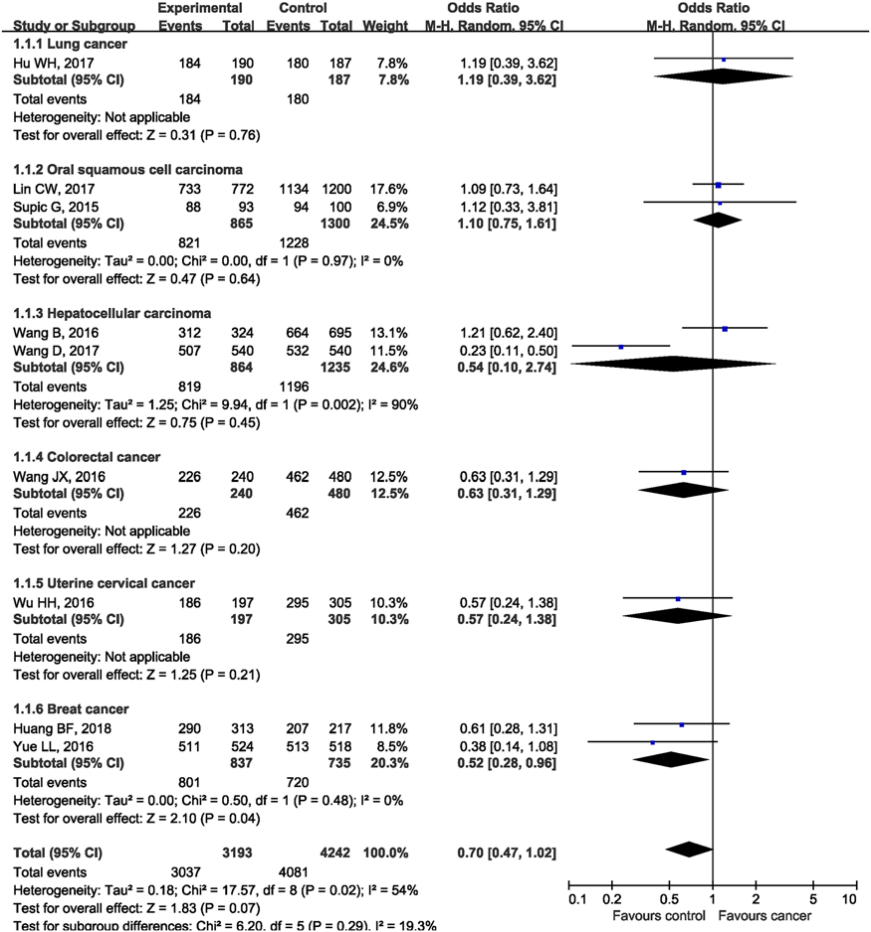
Forest plot of rs1045411 in *HMGB1* gene and risk of cancer using a dominant model: subgroup analysis by cancer type

**Figure 4:**
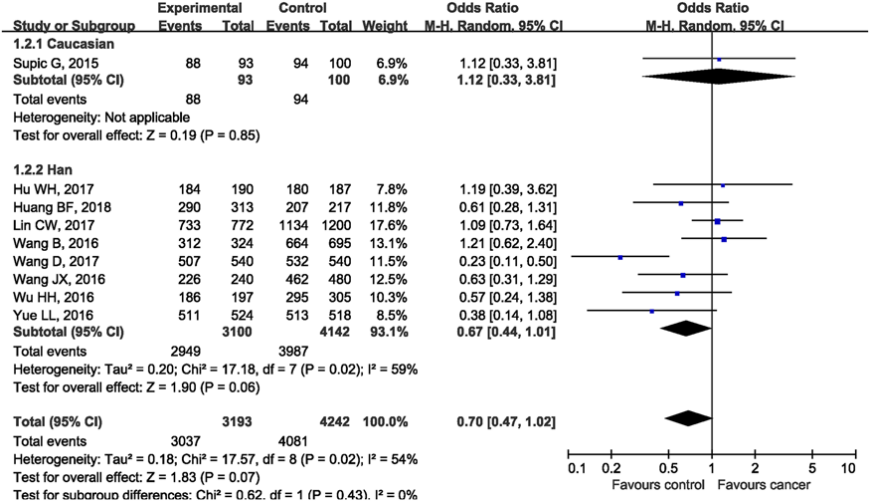
Forest plot of rs1045411 in HMGB1 gene and risk of cancer using a dominant model: subgroup analysis by ethnicity

In the subgroup analysis by the type of malignancies, no obvious differences in tumor risks could be found in HMGB1 rs1045411 polymorphism amongst any cancer type except for BC. The pooled ORs were 1.19 (95%CI: 0.39, 3.62; *P*=0.76), 1.10 (95%CI: 0.75, 1.61; *P*=0.64), 0.54 (95%CI: 0.10, 2.74; *P*=0.45), 0.63 (95%CI: 0.31, 1.29; *P*=0.20), 0.57 (95%CI: 0.24, 1.38; *P*=0.21), and 0.52 (95%CI: 0.28, 0.96; *P*=0.04) in LC, OSCC, HCC, CRC, UCC, and BC respectively (Figure 3). As for ethnicity, our results demonstrated that *rs1045411* polymorphism was not associated with risks of cancer amongst Hans (OR 0.67; 95%CI: 0.44, 1.01; *P*=0.06; Figure 4).

**Figure 5:**
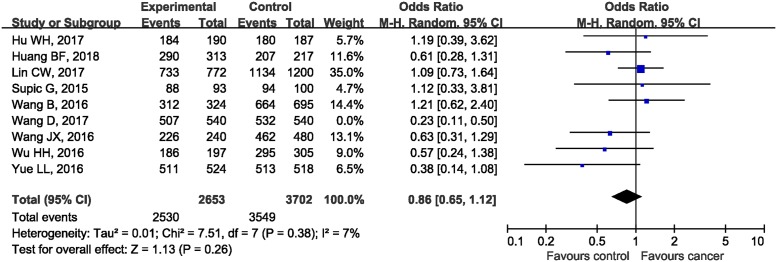
The leave-one-out sensitivity analysis

**Figure 6:**
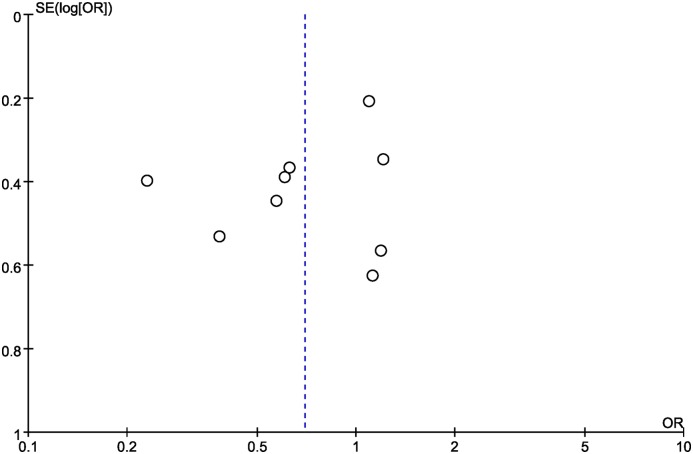
Funnel plot of rs1045411 in *HMGB1* gene and risk of cancer

We also performed the leave-one-out sensitivity analysis, and found that Wang et al. study [31] might contribute to the significant between-study heterogeneity. After the removal of Wang et al. study, the resulting heterogeneity across studies diminished from moderate heterogeneity (τ^2^ = 0.22; χ^2^ = 17.19; df = 7; *P*=0.02; *I^2^*= 59%) to low (τ^2^= 0.01; χ^2^ = 7.51; df = 7; *P*=0.38; *I^2^* = 7%; Figure 5). The funnel plot was visually symmetrical (Figure 6), the results of Begg’s test (z = 0.73, *P*=0.466) and Egger’s test (t = −1.18, *P*=0.276) also indicated no statistically significant publication bias.

## Discussion

To the best of our knowledge, this is the first systemic review and meta-analysis performed to access the correlation between HMGB1 polymorphism and cancer occurrence. Our study revealed no correlations between HMGB1 *rs1045411, rs1360485, rs1412125*, or *rs2249825* polymorphisms and cancer risks. Thereafter, we went further by conducting subgroup analysis of *rs1045411* based on cancer type and ethnicity stratification, which also indicated no correlations with cancer risks amongst all cancer types included except BC. No association was observed in the group of Hans or Caucasians.

HMGB1 is widely convinced as one of the most cancer-specific genes [45]. As a matter of fact, overexpression of HMGB1 is associated with each of the hallmarks of cancer as described by Hanahan and Weinberg [46], namely unlimited replicative potential, ability to develop blood vessels (angiogenesis), evasion of programmed cell death (apoptosis), self-sufficiency in growth signals, insensitivity to inhibitors of growth, inflammation, tissue invasion and metastasis [7]. Despite the fact that it is up-regulated in nearly every examined tumor, HMGB1 actually acts as a tumor suppressor and an oncogenic factor in tumorigenesis and cancer therapy, depending on complex conditions such as its diverse locations (namely, nuclear, cytosolic, membrane and extracellular HMGB1), binding partners, microenvironment, and different stages. Though complex, its role in cancer is indubitable.

The *rs1045411* polymorphism resides in the 3-flanking region, suggesting a role in mRNA stability as miRNAs can bind the 3′-UTR regions of mRNA transcripts and inhibit gene expression at the post-transcriptional level [31]. Meanwhile, probably due to a putative *miR-505* binding site, *rs1045411* and *rs1360485* 3′-UTR region variants showed strong linkage and were synergistically involved in the etiopathogenesis [28], which might explain why only *rs1045411* was connected with cancer risks in OR1 and 2 models in the current meta-analysis. We did not find statistically significant differences in cancer risks amongst carriers of this SNP variant compared with non-carriers, whether it was covered up by the counterbalance of its pleiotropic roles in cancer progression or reflected in diversified statistical strategies requires further investigation. However, there were trends toward an association with lower cancer susceptibility, which might become more apparent with a larger sample size.

Of note, studies included led to highly discrepant findings, as five of which revealed a protective role of CC+CT genotypes of *rs1045411* in cancer susceptibility [31–35], while the other four studies failed to replicate the initial findings [27–30]. This might be probably because of different geographical distributions of subjects, as all studies conducted amongst northern Hans [31,32,34] indicated the association between mutant alleles of *rs1045411* and an increased risk of malignancies, which was likely owing to environmental, lifestyle, or dietary factors. Since *rs1045411* polymorphism was closely correlated with an altered binding of *miR-505-5P* in the 3′-UTR of mRNA transcripts, *HMGB1* gene polymorphisms could emerge as a crucial player in cancer development through a post-transcriptional mechanism. However, when all available studies were combined together, the pooled ORs showed no association between *rs1045411* and risk of cancer in general (OR: 0.70, 95%CI: 0.47, 1.02; *P*=0.07), and the marginal overall effect is possibly due to limited number of studies included and number of subjects recruited in our selected studies. Therefore, we strongly call for additional studies with larger sample sizes and different ethnicities to confirm our findings.

Although *HMGB1* gene SNPs were found not related with risk of cancer based on the findings of our current meta-analysis, some of our included studies indicated that these SNPs could be associated with progression of cancer and overall survival outcomes. Wang et al. [30] indicated that HCC patients carrying at least one C allele at *rs1412125* showed a low risk of distant metastasis. Supic et al. [29] revealed that *rs2249825* and *rs3742305* polymorphisms might be associated with OSCC survival outcomes. Huang et al. [35] reported that patients with one G allele in *rs1360485* and *rs2249825* were likely to progress to T2 stage tumor and lymph node metastasis. C allele in *rs1412125* was found to be related with cancer progression, since Huang et al. reported that having one C allele increased the risk of pathologic grade 3 disease in HER2-enriched and triple-negative BC (TNBC). Furthermore, although multivariate analyses have demonstrated HMGB1 expression as an independent prognostic indicator of overall survival [47,48], the haplotype study has a greater statistical power and can be superior to individual SNP analysis for the detection of an association of the alleles with disease susceptibility [49]. Meanwhile, as more genetic loci are found, it may be possible to combine them into screening panels that provide more accurate prognostic data than an individual SNP alone. Moreover, there may be interactions between our studied locus and other genes involved in cancer progress as well.

According to the results of leave-one-out sensitivity analysis, research of Wang et al. [31] was found to contribute to the majority of the heterogeneity in our current study. After carefully scanning the full text, we found out that percentages of smokers and alcohol drinkers were significantly higher in patients than in controls (*P*<0.001) in this study, which might be confounding factors, as both of them were strikingly associated with the risk of HCC. Thus, the association between *rs1045411* and risk of cancer could be overstated because of the higher percentages of smokers and alcohol drinkers in the case group than control group. After eliminating this study, reevaluation of the remaining studies still revealed no statistically significant correlations, suggesting the robustness of the results in this meta-analysis.

Limited to the few scanty studies, our present study does have its limitations. Besides discussed above, as nine out of ten studies were conducted in Asia, our findings may not be representative of the general population, not to mention samples of cases recruited in each cancer type. Moreover, the association between SNPs in HMGB1 and cancer risks may be affected by other confounders including smoking status, drinking status as well as gender and ages, but we are currently unable to perform further stratified analysis based on these confounding factors due to the incomplete raw data reported by included studies. Under such condition, large-scale studies, involving haplotype analysis as well as adjustments of potential confounding factors, are required to further elucidate the exact role of HMGB1 and its variants in cancer.

## Conclusion

Our current systemic review and meta-analysis mainly focussed on associations between HMGB1 *rs1045411, rs1360485, rs1412125*, or *rs2249825* polymorphisms and cancer susceptibility, we went further by conducting subgroup analysis based on cancer type and ethnic groups of subjects, and revealed no statistical differences between rs1045411 polymorphism and cancer risk amongst LC, OSCC, HCC, CRC, and UCC. Subgroup analysis by ethnicity showed no association of *rs1045411* and risk of cancer in Hans or Caucasians. Although our results appeared to be obscure statistically, which might postpone the potential application of HMGB1 as a therapeutic target as well as a prognosis predictor, reasonable confidence should be given to the null association between HMGB1 polymorphisms and risk of cancer. We strongly call for further investigations to lift the veil of the underlying mechanisms involved in the relationship between HMGB1 SNPs and cancer risk.

## Abbreviations

BC: breast cancer
CRC: colorectal cancer
HCC: hepatocellular carcinoma
HMGB1: high mobility group box 1
HWE: Hardy–Weinberg equilibrium
LC: lung cancer
NOS: Newcastle–Ottawa Scale
OR: odds ratio
OSCC: oral squamous cell carcinoma
Rb: retinoblastoma protein
UCC: uterine cervical cancer
95%CI: 95% confidence interval

## References

[1] Bustin M. (2001) Revised nomenclature for high mobility group (HMG) chromosomal proteins. Trends Biochem. Sci. 26, 152–153 10.1016/S0968-0004(00)01777-1 11246012

[2] Muller S., Ronfani L. and Bianchi M.E. (2004) Regulated expression and subcellular localization of HMGB1, a chromatin protein with a cytokine function. J. Intern. Med. 255, 332–343 10.1111/j.1365-2796.2003.01296.x 14871457

[3] Wang H., Zhu S., Zhou R., Li W. and Sama A.E. (2008) Therapeutic potential of HMGB1-targeting agents in sepsis. Expert Rev. Mol. Med. 10, e32 10.1017/S1462399408000884 18980707 PMC2586610

[4] Kang R., Chen R., Zhang Q. (2014) HMGB1 in health and disease. Mol. Aspects Med. 40, 1–116 10.1016/j.mam.2014.05.001 25010388 PMC4254084

[5] Polanska E., Dobsakova Z., Dvorackova M., Fajkus J. and Stros M. (2012) HMGB1 gene knockout in mouse embryonic fibroblasts results in reduced telomerase activity and telomere dysfunction. Chromosoma 121, 419–431 10.1007/s00412-012-0373-x 22544226

[6] Kang R., Livesey K.M., Zeh H.J., Loze M.T. and Tang D. (2010) HMGB1: a novel Beclin 1-binding protein active in autophagy. Autophagy 6, 1209–1211 10.4161/auto.6.8.13651 20935509

[7] Venereau E., De Leo F., Mezzapelle R., Careccia G., Musco G. and Bianchi M.E. (2016) HMGB1 as biomarker and drug target. Pharmacol. Res. 111, 534–544 10.1016/j.phrs.2016.06.031 27378565

[8] Andersson U. and Tracey K.J. (2011) HMGB1 is a therapeutic target for sterile inflammation and infection. Annu. Rev. Immunol. 29, 139–162 10.1146/annurev-immunol-030409-101323 21219181 PMC4536551

[9] Todorova J. and Pasheva E. (2012) High mobility group B1 protein interacts with its receptor RAGE in tumor cells but not in normal tissues. Oncol. Lett. 3, 214–218 10.3892/ol.2011.459 22740883 PMC3362388

[10] Kang R., Tang D., Schapiro N.E. (2014) The HMGB1/RAGE inflammatory pathway promotes pancreatic tumor growth by regulating mitochondrial bioenergetics. Oncogene 33, 567–577 10.1038/onc.2012.631 23318458 PMC3795800

[11] Zhang Z., Wang M., Zhou L. (2015) Increased HMGB1 and cleaved caspase-3 stimulate the proliferation of tumor cells and are correlated with the poor prognosis in colorectal cancer. J. Exp. Clin. Cancer Res. 34, 51 10.1186/s13046-015-0166-1 25986235 PMC4446854

[12] Volp K., Brezniceanu M.L., Bosser S. (2006) Increased expression of high mobility group box 1 (HMGB1) is associated with an elevated level of the antiapoptotic c-IAP2 protein in human colon carcinomas. Gut 55, 234–242 10.1136/gut.2004.062729 16118352 PMC1856519

[13] Zhang X., Yu J., Li M., Zhu H., Sun X. and Kong L. (2016) The association of HMGB1 expression with clinicopathological significance and prognosis in Asian patients with colorectal carcinoma: a meta-analysis and literature review. Onco. Targets Ther. 9, 4901–4911 10.2147/OTT.S105512 27540303 PMC4982502

[14] Chen M., Liu Y., Varley P. (2015) High-mobility group box 1 promotes hepatocellular carcinoma progression through miR-21-mediated matrix metalloproteinase activity. Cancer Res. 75, 1645–1656 10.1158/0008-5472.CAN-14-2147 25720799 PMC4401643

[15] Zhang L., Han J., Wu H. (2014) The association of HMGB1 expression with clinicopathological significance and prognosis in hepatocellular carcinoma: a meta-analysis and literature review. PLoS ONE 9, e110626 10.1371/journal.pone.0110626 25356587 PMC4214718

[16] Lee H.J., Kim J.Y., Song I.H. (2015) High mobility group B1 and N1 (HMGB1 and HMGN1) are associated with tumor-infiltrating lymphocytes in HER2-positive breast cancers. Virchows Arch. 467, 701–709 10.1007/s00428-015-1861-1 26445971

[17] Nguyen A.H., Detty S.Q. and Agrawal D.K. (2017) Clinical implications of high-mobility group box-1 (HMGB1) and the receptor for advanced glycation end-products (RAGE) in cutaneous malignancy: a systematic review. Anticancer Res. 37, 1–7 10.21873/anticanres.11282 28011467

[18] Wang H., Li Z., Sun Y. (2015) Relationship between high-mobility group box 1 overexpression in ovarian cancer tissue and serum: a meta-analysis. Onco. Targets Ther. 8, 3523–3531 26664135 10.2147/OTT.S93357 PMC4669932

[19] Jube S., Rivera Z.S., Bianchi M.E. (2012) Cancer cell secretion of the DAMP protein HMGB1 supports progression in malignant mesothelioma. Cancer Res. 72, 3290–3301 10.1158/0008-5472.CAN-11-3481 22552293 PMC3389268

[20] Wu T., Zhang W., Yang G. (2016) HMGB1 overexpression as a prognostic factor for survival in cancer: a meta-analysis and systematic review. Oncotarget 7, 50417–50427 27391431 10.18632/oncotarget.10413 PMC5226592

[21] Kang R., Zhang Q., Zeh H.J. III, Lotze M.T. and Tang D. (2013) HMGB1 in cancer: good, bad, or both? Clin. Cancer Res. 19, 4046–4057 10.1158/1078-0432.CCR-13-0495 23723299 PMC3732559

[22] Jiao Y., Wang H.C. and Fan S.J. (2007) Growth suppression and radiosensitivity increase by HMGB1 in breast cancer. Acta Pharmacol. Sin. 28, 1957–1967 10.1111/j.1745-7254.2007.00669.x 18031610

[23] Wang L.L., Meng Q.H., Jiao Y. (2012) High-mobility group boxes mediate cell proliferation and radiosensitivity via retinoblastoma-interaction-dependent and -independent mechanisms. Cancer Biother. Radiopharm. 27, 329–335 10.1089/cbr.2012.1199 22655796 PMC3375072

[24] Huang S. (2013) Genetic and non-genetic instability in tumor progression: link between the fitness landscape and the epigenetic landscape of cancer cells. Cancer Metastasis Rev. 32, 423–448 10.1007/s10555-013-9435-7 23640024

[25] Duijf P.H. and Benezra R. (2013) The cancer biology of whole-chromosome instability. Oncogene 32, 4727–4736 10.1038/onc.2012.616 23318433

[26] Ke S., Zhou F., Yang H. (2015) Downregulation of high mobility group box 1 modulates telomere homeostasis and increases the radiosensitivity of human breast cancer cells. Int. J. Oncol. 46, 1051–1058 10.3892/ijo.2014.2793 25501936

[27] Hu W., Liu P.Y., Yang Y.C. (2017) Association of HMGB1 gene polymorphisms with lung cancer susceptibility and clinical aspects. Int. J. Med. Sci. 14, 1197–1202 10.7150/ijms.20933 29104475 PMC5666552

[28] Lin C.W., Chou Y.E., Yeh C.M., Yang S.F., Chuang C.Y. and Liu Y.F. (2017) A functional variant at the miRNA binding site in HMGB1 gene is associated with risk of oral squamous cell carcinoma. Oncotarget 8, 34630–34642 28423715 10.18632/oncotarget.16120 PMC5470997

[29] Supic G., Kozomara R., Zeljic K. (2015) HMGB1 genetic polymorphisms in oral squamous cell carcinoma and oral lichen planus patients. Oral Dis. 21, 536–543 10.1111/odi.12318 25639284

[30] Wang B., Yeh C.B., Lein M.Y. (2016) Effects of HMGB1 polymorphisms on the susceptibility and progression of hepatocellular carcinoma. Int. J. Med. Sci. 13, 304–309 10.7150/ijms.14877 27076788 PMC4829544

[31] Wang D., Qi X., Liu F. (2017) A multicenter matched case-control analysis on seven polymorphisms from HMGB1 and RAGE genes in predicting hepatocellular carcinoma risk. Oncotarget 8, 50109–50116 28187002 10.18632/oncotarget.15202 PMC5564833

[32] Wang J.X., Yu H.L., Bei S.S. (2016) Association of HMGB1 gene polymorphisms with risk of colorectal cancer in a Chinese population. Med. Sci. Monit. 22, 3419–3425 10.12659/MSM.896693 27665685 PMC5040220

[33] Wu H.H., Liu Y.F., Yang S.F. (2016) Association of single-nucleotide polymorphisms of high-mobility group box 1 with susceptibility and clinicopathological characteristics of uterine cervical neoplasia in Taiwanese women. Tumour Biol. 10.1007/s13277-016-5408-0 27704361

[34] Yue L., Zhang Q., He L. (2016) Genetic predisposition of six well-defined polymorphisms in HMGB1/RAGE pathway to breast cancer in a large Han Chinese population. J. Cell. Mol. Med. 20, 1966–1973 10.1111/jcmm.12888 27241711 PMC5020633

[35] Huang B.F., Tzeng H.E., Chen P.C. (2018) HMGB1 genetic polymorphisms are biomarkers for the development and progression of breast cancer. Int. J. Med. Sci. 15, 580–586 10.7150/ijms.23462 29725248 PMC5930459

[36] Jiang M., Li X., Quan X., Li X. and Zhou B. (2018) Single nucleotide polymorphisms in HMGB1 correlate with lung cancer risk in the northeast Chinese Han population. Molecules 23, 10.3390/molecules23040832 PMC6017634 29617336

[37] Lee J., Choi J., Chung S. (2017) Genetic predisposition of polymorphisms in HMGB1-related genes to breast cancer prognosis in Korean women. J. Breast Cancer 20, 27–34 10.4048/jbc.2017.20.1.27 28382092 PMC5378577

[38] Bao G., Qu F., He L. (2016) Prognostics ignificance of Tag SNP rs1045411 in HMGB1 of the aggressive gastric cancer in a Chinese population. PLoS ONE 11, e0154378 10.1371/journal.pone.0154378 27116470 PMC4845981

[39] Lawson T. and Gannett P. (1989) The mutagenicity of capsaicin and dihydrocapsaicin in V79 cells. Cancer Lett. 48, 109–113 10.1016/0304-3835(89)90045-1 2684392

[40] Moher D., Liberati A., Tetzlaff J., Altman D.G. and Group P. (2009) Preferred reporting items for systematic reviews and meta-analyses: the PRISMA statement. PLoS Med. 6, e1000097 10.1371/journal.pmed.1000097 19621072 PMC2707599

[41] Wells G.A., Shea B., O’Connell D. The Newcastle-Ottawa Scale (NOS) for assessing the quality of nonrandomised studies in meta-analyses. http://www.ohri.ca/programs/clinical_epidemiology/oxford.asp

[42] Thakkinstian A., McElduff P., D’Este C., Duffy D. and Attia J. (2005) A method for meta-analysis of molecular association studies. Stat. Med. 24, 1291–1306 10.1002/sim.2010 15568190

[43] Mantel N. and Haenszel W. (1959) Statistical aspects of the analysis of data from retrospective studies of disease. J. Natl. Cancer Inst. 22, 719–748 13655060

[44] Egger M., Davey Smith G., Schneider M. and Minder C. (1997) Bias in meta-analysis detected by a simple, graphical test. BMJ 315, 629–634 10.1136/bmj.315.7109.629 9310563 PMC2127453

[45] Martinotti S., Patrone M. and Ranzato E. (2015) Emerging roles for HMGB1 protein in immunity, inflammation, and cancer. Immunotargets Ther. 4, 101–109 27471716 10.2147/ITT.S58064 PMC4918250

[46] Hanahan D. and Weinberg R.A. (2000) The hallmarks of cancer. Cell 100, 57–70 10.1016/S0092-8674(00)81683-9 10647931

[47] Ueda M., Takahashi Y., Shinden Y. (2014) Prognostic significance of high mobility group box 1 (HMGB1) expression in patients with colorectal cancer. Anticancer Res. 34, 5357–5362 25275029

[48] Xu Y., Chen Z., Zhang G. (2015) HMGB1 overexpression correlates with poor prognosis in early-stage squamous cervical cancer. Tumour Biol. 36, 9039–9047 10.1007/s13277-015-3624-7 26084608

[49] Shifman S., Bronstein M., Sternfeld M. (2002) A highly significant association between a COMT haplotype and schizophrenia. Am. J. Hum. Genet. 71, 1296–1302 10.1086/344514 12402217 PMC378567

